# Fidelity, not adaptation, is essential for implementation

**DOI:** 10.3389/frhs.2025.1575179

**Published:** 2025-04-25

**Authors:** Dean L. Fixsen

**Affiliations:** Active Implementation Research Network, Inc., Albuquerque, NM, United States

**Keywords:** fidelity, essential components, science, implementation, scaling

## Abstract

Fidelity is not yet a requirement when developing an evidence-based innovation or when attempting to use an innovation in typical settings. Currently, users are encouraged to adapt innovations to fit existing practitioner skills and organization situations. Instead of adapting innovations, the essential components of an innovation need to be established in the original research and the essential components need to be used in practice with the support of implementation processes so that promised outcomes can be realized. Fidelity is an assessment of the presence and strength of the essential components that define the independent variable (the innovation) and is directly linked to outcomes. A test of any fidelity assessment is a high correlation (0.70+) with outcomes. The functional relationship between fidelity and outcomes ensures that the essential components are effective and ensures that a reliable fidelity assessment is available. Implementation is the planned process of putting something into effect. Evidence that an innovation has been put into effect is provided by the fidelity assessment. High fidelity scores indicate that the essential components of the innovation are in place and good outcomes are expected. A test of any planned process is fidelity of the use of the innovation. At present fidelity assessments are missing or inadequate and, therefore, there is a notable lack of evidence that an independent variable is present.

## Introduction

Fidelity often is viewed as optional when developing an evidence-based innovation or when attempting to use evidence-based innovations to benefit individuals and society (1). Instead of using an innovation as intended (with fidelity), a “science of adaptation” has been proposed (2) for the process of adapting innovations. An alternative view is that fidelity is essential. Fidelity is integral to the definition of an innovation, is essential when developing an evidence-based innovation, and is the standard to meet when using an innovation (3–5). Instead of adapting the innovation, the goal of implementation is to change practitioner, organization, and system behavior so that innovations can be used with fidelity and good outcomes. What is needed is a science of implementation, not a science of adaptation.

The essential role of fidelity assessment in science, in implementation, and in practice is summarized in this paper.

## Fidelity in science

In science, fidelity is an assessment of the presence and strength of the independent variable in experiments to establish if-then relationships—if “this” is done, then “that” happens consistently. Experiments that test if-then predictions provide the evidence that is the foundation for any science (6, 7).

An experiment provides evidence of a functional relationship, that is, one variable systematically affects another. “A scientist (and the audience) must be assured that the implementation factor (if this: the independent variable) is present and at sufficient strength so that the results (then that: the dependent variable) reasonably can be attributed to the implementation factor. For interaction-based innovations, the independent variable must be measured repeatedly throughout an experiment with the same accuracy and care as the dependent variable” (8). Thus, the function of fidelity in science is to assure the scientific community that the independent variable “is there”—we did “this” with a known level of strength, and “that” outcome was produced (or not).

In science, any credible experiment to test if-then relationships provides (a) a clear description of the essential components of the independent variable, (b) indicators that the essential components of the independent variable are present and at sufficient strength to be tested, and (c) evidence that outcomes are directly attributable to the essential components of the independent variable. The direct tie between essential components and indicators of the presence and strength of those components means that fidelity always is specific to an innovation [referred to as program differentiation (9, 10)]. Fidelity provides evidence that the essential components of *this* innovation are present and at sufficient strength to have an impact. An experiment provides evidence that the essential components are highly related to the outcomes (if this, then that). In science, outcomes cannot be attributed to something that is not there, although in practice specious attributions are not uncommon (11).

Thus, a fidelity assessment is a requirement for any research to develop an evidence-based innovation. The scientific community needs to know what “it” is, and “it” needs to be assessed to assure the scientific community that “it” was present and used as described (12). While there are several ways to develop an assessment (9, 13), the test of any fidelity assessment is its relationship with (i.e., prediction of) intended outcomes. For example, a positive or negative correlation of 0.70 or better would indicate that the essential components have been adequately identified and assessed, and they are effective [a correlation coefficient greater than 0.7 is considered strong; (14)]. A correlation of 0.70 or better explains 50% or more of the variance in outcomes. If there is a strong relationship, then the innovation would be “worth doing” with high fidelity so that socially significant outcomes could be achieved.

The fidelity-outcome relationship was tested in a study to develop a fidelity assessment for cognitive behavioral therapy for insomnia. The study found a 0.30 correlation between therapist fidelity scores and treatment outcomes, explaining about 10% of the variability in outcomes (15). In another example, the Washington State Institute for Public Policy (16) examined fidelity of the use of Functional Family Therapy (FFT) (17–19). The 12-month post treatment delinquency outcomes were assessed for referred youths in the 427 families treated by 25 FFT therapists. An analysis of the data found a −0.61correlation between the fidelity of the therapists’ use of FFT and youth recidivism, explaining about 36% of the variability in delinquency outcomes. A quintile analysis found 8% recidivism among the youths in families treated by FFT therapists in the top 20% of fidelity scores, and 34% recidivism for the youths in families treated by FFT therapists in the bottom 20% of fidelity scores. When FFT was present and at sufficient strength (top 20%), outcomes for youths, families, and society were 4X better. The fidelity-outcome relationship provides evidence that the essential components have been (more or less) adequately specified and are effective—important information for potential users.

An, Dusing (4) recommend setting a standard for fidelity of the use of the independent variable (e.g., 80%) that must be met *before* an experiment begins. For example, Tofail, Fernald (20) conducted a study of individual and combined water, sanitation, handwashing and child nutrition interventions delivered by community health workers (CHWs) to pregnant women and their infants in 4,169 households in Bangladesh. Three months after initiating the experiment, assessments indicated fidelity was low (30%–60% range). Extra support for the CHWs was provided, fidelity scores improved (86%–93% range), and only then was the experiment conducted (21). Fidelity scores remained high (22) and ensured the continued presence and strength of the multifaceted independent variable. As a result, the researchers provided a true test of the combined water, sanitation, handwashing and child nutrition interventions—“it” was there, and outcomes could be evaluated and attributed to “it”.

In perhaps the most elegant implementation experiment ever conducted (23), a 2 × 2 design was carried out in 14 rural Appalachian counties to test the effects of organization support on implementation success. The design included 2 factors: (a) the random assignment of delinquent youth within each county to a multisystemic therapy (MST) program or usual services and (b) the random assignment of counties to the ARC (Availability, Responsiveness, and Continuity) organizational intervention. MST teams were developed using established implementation protocols for therapist selection, training, and supervision, and therapist fidelity was regularly assessed. ARC specialists were trained and supervised by the ARC developers at the University of Tennessee and fidelity was monitored with on-site observation and activity logs. The combination of evidence-based treatment (MST) and facilitative organization support (ARC) produced the best outcomes for delinquent youths. Fidelity assessments provided assurance that each complex evidence-based innovation (MST and ARC) was there and at sufficient strength to conduct a credible test of their individual and combined effects in multiple counties over four years.

Fidelity assessment requires attention to the essential components, what “it” is and the key indicators of the presence and strength of “it.” With a required fidelity assessment, high fidelity [at least 80% according to An, Dusing (4)] ensures the independent variable was present and at sufficient strength to provide a valid test of its effects. If fidelity is not high, or not strongly related to outcomes, the time to correct the problem is during the original research. Have the essential components been identified adequately? Have the essential components been measured adequately? If the identification and assessment of the essential components are adequate, do the essential components need to be improved (discarded, changed) to produce better outcomes? Deferring the solution to these fundamental questions puts the onus on potential users who want to benefit others. However, inadequate science plus local adaptation likely will not equal socially significant benefits for intended beneficiaries.

In science, fidelity is directly linked to the essential components of an innovation, fidelity provides indicators of the presence and strength of the essential components, and fidelity is highly correlated with outcomes. With a firm commitment to fidelity, “this” is defined, “that” is known, and “this” and “that” are improvable as the science evolves.

## Fidelity in implementation

Implementation is the planned process for putting something into effect (24–27). Thus, a “planned process” is the implementation independent variable (if this), and “putting something into effect” is the implementation dependent variable (then that). In a science of implementation, fidelity assessment is doubly important: (a) it provides an indication of the presence and strength of the implementation independent variable (the planned process), and (b) it provides an indication that the essential components of something have been put into effect (the implementation dependent variable).

What is the “something” (i.e., defined by the essential components) and how do we know it was “put into effect” (i.e., assessed with a measure of fidelity)? In a science of implementation, innovation fidelity always is a dependent variable, an outcome of effective implementation processes (25, 28). Thus, innovation fidelity has a dual role as an implementation dependent variable and an innovation independent variable. This is a common feature in nested systems where one component, simultaneously, is a singular unit and a part of a larger whole (29, 30). In effect, every independent variable at one level is a dependent variable at the next level (8, 31).

Logically, (a) implementation specialists engage in high fidelity implementation processes, (b) so that practitioners will provide high fidelity services, (c) so that recipients will benefit. Proctor, Bunger (32) found that studies related to this predicted relationship are not common. In their analysis of 400 studies of implementation outcomes, Proctor, Bunger (32) found 22 studies relating implementation outcomes with service outcomes, with 2 of those studies focusing on fidelity. Similarly, in a search for repeated measures of implementation variables, Fixsen, Van Dyke (33) found 17 articles that assessed innovation fidelity two or more times in the course of an experiment. Thus, although innovation fidelity is recognized as an implementation dependent variable, it is not studied frequently in implementation science.

Assessing innovation fidelity immediately directs attention to implementation processes. If fidelity is low, instead of adapting the innovation, what implementation processes can be used to prepare practitioners to use 80% or more of the innovation's essential components consistently? What implementation processes can be used to help an organization change to effectively support practitioners’ use on the innovation? What implementation processes can be used to help leaders and managers provide leadership for change, or change policies and procedures to support the continuing and effective use of an innovation? These are proximal implementation variables and have an immediate effect on the use (or nonuse) of any innovation (31, 34–37). If fidelity is high and outcomes are poor, the next right step is to conduct experiments to re-examine the putative essential components (try again, back to fidelity in science). If fidelity is not assessed at all, there are no next right steps and there is no prompt to improve the innovation or attend to implementation processes.

Fidelity is a necessary and critical link between implementation processes and ultimately achieving intended outcomes. In this process, potential users are not encouraged to adapt the very things (the essential components) that produce desired outcomes. Improved fit almost always requires changing practitioner behavior and organization and system behavior so that the essential components of an innovation can be used successfully (38–40). The processes for changing practitioner, organization, and system behavior are implementation independent variables. Practitioners, organizations, and systems that attempt to use innovations without making any changes in their ways of work—doing the same thing again and again and expecting different results—are certain to fail.

Implementation independent variables are “planned processes” that have an immediate and longer-term effect on the use (or nonuse) of any innovation. In a science of implementation, innovation fidelity is always a dependent variable for implementation independent variables, the test that “something has been put into effect”.

## Preparing for everyday use

An innovation should not be expected to be usable in general practice until it has undergone usability testing (39, 41). For multifaceted and complex interaction-based innovations in human services, well defined and operationalized essential components likely will be incomplete. And measurement of each component may not be feasible given the sometimes private or fast paced nature of human interactions. Even so, scientists must make every effort to define the essential components and find credible ways to assess their presence and strength. The test is that fidelity and outcomes are highly related.

Usability testing is a well-established, systematic approach to “working out the bugs” in any complex program or system intended for general use (39, 42–45). Usability testing is based on plan-do-study-act-cycle (PDSAC) logic (46–48). In usability testing, a small number of participants (*n*∼5) attempt to use an innovation in each Cycle. The Plan is to use the essential components of the innovation. Each participant then Does the plan. The testing team Studies the results: did the individuals Do the Plan (fidelity) and to what extent were intended outcomes achieved? The testing team Acts on the information by changing the innovation and modifying the fidelity assessment to reflect the changes. The participants in the next group then use the essential components of the improved innovation (the new Plan) in the next Cycle. This process is repeated until the innovation (the Plan) is improved to the point that intended outcomes are achieved reliably and are highly correlated with fidelity scores. Three to 5 cycles may be sufficient to detect and correct 80% or more of the errors in the original Plan (49).

In usability testing, with the evolving fidelity assessment as the standard, factors that negatively impact achieving the standard can be detected and corrected without compromising the function—if this, then that. Fidelity is the “bug detector,” an indication that something is getting in the way of doing what is required (i.e., the essential components) to produce desired outcomes. With each iteration in usability testing, “the pool of effective methods expands to incorporate effective responses to what previously was unanticipated. The expanded methods then can benefit a greater proportion of the variations encountered in communities, service settings, and organizations” (8). The result is a set of robust and generalizable implementation methods that support the full and effective (high fidelity) use of the innovation so that desired outcomes can be achieved consistently. Of course, usability testing must be done with fidelity to be effective (47, 50).

Successive groups of 5 users to detect and correct errors is recommended by the developers of usability testing (42, 43). Barker, Reid (51) advocate usability testing with increasing numbers of users to ensure exposure to an increasing range of real-life situations. Barker, Reid (51) provide examples where “the rate of expansion can be exponential (i.e., not linear) by a multiple of 5… (e.g., 1–5–25–125–625, etc.).” At each level, external validity is strengthened as revised methods are established to resolve newly exposed problems before moving to the next level. Appropriate adaptations become a part of the definition of an innovation as exposure to new problems invites new solutions so that the problems are solved, and outcomes are achieved. In this way, “the pool of effective methods” is expanded to include constructive responses to variations related to culture, race, gender, socio-economic conditions, geography, seasons, territorial conflicts, local contexts, and so on. Usability testing also can detect the limits of the use of the innovation, the conditions under which the essential components do not produce the outcomes found under other conditions. For example, a well-defined innovation for youths adjudicated as serious delinquents does not produce similar outcomes for youths with severe mental health problems (52). Usability testing is work for the scientific community.

Unfortunately, usability testing is not common. Instead of doing the work required to establish evidence-based innovations and evidence-based implementation processes, the challenges are passed to potential users. The notion of tailoring asks thousands of potential users of an innovation to do the work the developers were unwilling or unable to do—specify the essential components and provide indicators to assess their presence and strength. Increasing uncertainty and variability by tailoring, and shifting responsibility to local users will not solve the problems confronting human services (53–58).

For researchers and program developers, usability testing is an extra step to establish the internal validity and external validity of an innovation before it is released for general use. Extra steps are not unusual in science. Early in the evidence-based innovation movement, concerns about the “evidence” led to CONSORT guidelines related to the quality of randomized controlled trials (RCTs) [described by Altman, Schulz (59)]. As Eldridge, Ashby (60) stated, internal validity can be strengthened with “good design, conduct, and analysis of the trial, with minimal bias, …and sufficient sample size.” In the seminal CONSORT paper, there is no mention of fidelity, no mention of an independent variable, and only 3 uses of the word “intervention” where researchers were encouraged to “suggest a plausible explanation for how the intervention under investigation might work” (p. 667). With regard to evidence, Altman, Schulz (59) summarized CONSORT by stating, “Reports of RCTs should be written with… close attention to minimizing bias. Readers should not have to speculate; the methods used should be transparent, so that readers can readily differentiate trials with unbiased results from those with questionable results. Sound science encompasses adequate reporting, and the conduct of ethical trials rests on the footing of sound science”.

Currently, the growing science to service gap has led to the guidelines outlined in this paper regarding the internal and external validity of the innovation itself. For evidence-based innovations, it is not enough to have rigorously derived “evidence,” the “innovation” also must be well defined. The CONSORT statement can be paraphrased: Readers should not have to speculate; with a usable innovation readers can readily differentiate innovations with clearly specified essential components from those with questionable components. Sound science encompasses adequate description and measurement of essential components, and the conduct of ethical usability testing rests on the footing of sound science.

Avoiding fidelity assessment is avoiding learning what we need to know to create evidence-based implementation processes. Encouraging users to adapt methods may increase their acceptability to users but not their benefits to recipients. In science and in practice, changing methods changes outcomes.

## Fidelity in practice

Fidelity sets a minimum standard, the least that users need to do in order to say they are “using” an innovation (61–64). Taking anything to a useful scale requires increasing standardization of innovations, implementation supports, and operating environments to reduce unwanted (potentially harmful) sources of variability (5, 65–68). Achieving a useful standard requires greater attention to innovation development and implementation methods so that thousands of practitioners can use evidence-based innovations with fidelity to provide benefits to whole populations.

For example, smallpox was eradicated globally using surveillance teams and containment teams (69). The teams were the innovation independent variables in the efforts to eradicate smallpox for the population on Earth. Foege (70) and colleagues, working in rural Africa in the 1960s, developed containment teams to isolate and treat the infected and inoculate the exposed. In their early work (i.e., usability testing) they relied on local networks to identify newly infected people. Foege and colleagues found that local networks often missed new outbreaks of smallpox. The response was to establish surveillance teams to systematically and reliably find those who were infected. As they gained experience and collected more data, they established protocols (i.e., operationalized essential components) for each team and “increased the pool of effective methods” based on effectiveness and efficiency outcomes. Foege (70) recounts the challenges faced in India in the 1970s (population over 600 million in 27 states). National, state, regional, and local implementation supports were established so that surveillance teams and containment teams could be created and sustained in every state and each “block” of 100,000 people so that they could reach all the people in each urban neighborhood and each village. Foege's story is about increasing the specificity of protocols, increasing the frequency of fidelity, process, and outcome monitoring, and increasing the reliance on data so that effectiveness and efficiency of the surveillance teams and containment teams were immediately and continually improved.

“The strategy for smallpox eradication did not change from country to country, but the local culture determined which tactics were most useful. Only the specific locality can provide information on who is sick, who is hiding from the vaccinators, when people are available for vaccination, how to hire watch guards, or how to secure the cooperation of the community. In all cultures, an approach of respect for local customs is needed” (70). Thus, “methods to respect local customs” was one of the essential components of the standard protocol for a surveillance team.

Surveillance teams and containment teams were not adapted to fit local contexts. Instead, the protocols for implementation processes, teams, fidelity assessments, and outcome assessments were standardized to reduce variability and error and improve outcomes. In one month, surveillance teams searched 140,000 villages in one state using standard protocols. The surveillance teams did not stop 140,000 times to figure out how the essential components of surveillance teams should be adapted. Instead, the surveillance teams used the standard protocols 140,000 times with high fidelity to accomplish the intended outcomes.

In human services, standard fidelity assessments for evidence-based programs have been developed and used for many years across many contexts. For example, the Teaching-Family Model began in 1967 as a group home residential treatment program for delinquent youths. After the first replication attempt failed in 1971, a fidelity assessment was developed, tested, and refined (71–73). Early work (i.e., usability testing) provided evidence that practitioner development was insufficient for sustainability (74). This led to developing Teaching-Family organizations with implementation teams built into each organization, and sustainability (5+ years) improved from 15% (*n* = 84 group homes) to 83% (*n* = 219 group homes) (75). The Teaching-Family fidelity assessment has been used in every Teaching-Family treatment service setting (i.e., group home, foster family, homebased, or school-based) for over 50 years (75–77).

Fidelity assessment has been used on a large scale by Positive Behavior Interventions and Supports (PBIS). PBIS is an evidence-based multifaceted whole school intervention developed to reduce student discipline problems and improve academic outcomes (78, 79). Fidelity assessments were developed and tested (80), revised (81), and used as PBIS expanded to over 30,000 of the 100,000 schools in the US. McIntosh, Mercer (82) analyzed PBIS fidelity data each year for 5 years for 5,331 schools located in 1,420 school districts in 37 states. They found evidence of shifting patterns of fidelity they categorized as sustainers, slow starters, late abandoners, and rapid abandoners. Similarly, Motivational Interviewing (83) is in widescale use with standard assessments of fidelity and supervision (84–86).

Forgatch, Patterson (87) used elements of two previously developed observation methods and, “through an iterative process” (i.e., usability testing), established new protocols for observation of the Parent Management Training-Oregon (PMTO) program essential components. The PMTO fidelity assessment has been used in the national scale up of PMTO in Norway (88). The first “generations” of PMTO practitioners learned from the original researchers, became certified PMTO therapists, and then learned to carry out the implementation supports with fidelity in Norway and beyond (89). A 10-year assessment of implementation capacity development (i. e., recruitment, training, coaching, fidelity assessments, administration, leadership, etc.) for scale up in Norway found continued high fidelity use of essential implementation components, high fidelity use of essential innovation components by “generations” of practitioners, and sustained benefits for children and families (5, 90).

These examples of long-term and functional fidelity are not typical at this stage of the evidence-based movement. Reviews of the literature over the past several decades consistently find that fidelity is not measured in the majority of outcome studies, and repeated use of any fidelity measure is even less common (91, 92). When it is present, fidelity assessment has focused on form (e.g., frequency, duration, dosage, participation) rather than function (i.e., fidelity scores are highly correlated with desired outcomes). Eventually, with a high correlation as a benchmark, the wide variety of “fidelity measures” will be replaced by functional ones that can be relied on by potential users. In the meantime, potential users will continue to cope with innovations with uncertain essential components and questionable fidelity assessments, and scaling to achieve socially significant benefits will remain an aspirational goal.

Fortunately, there are fidelity assessments in everyday use in many sectors to ensure the expanded and sustained use of evidence-based innovations (21, 31, 52, 93–99). In everyday use, the essential components of an innovation must be present (used with fidelity) so that their outcomes can be produced.

## Summary

To close the science to service gap and produce benefits to recipients of those services (i.e., the goals of implementation in human services), we need to get the science right, right from the beginning. For any innovation or implementation independent variable, scientists must specify the essential components, provide indicators (fidelity measures) of the presence and strength of those essential components, and provide evidence that outcomes are strongly associated with the strength of those essential components. For any use of an innovation or implementation independent variable (practice, program, or policy), fidelity is the standard to achieve so that desired outcomes can be realized. With a firm commitment to fidelity, “this” is defined, “that” is known, and “this” and “that” are improvable as the science evolves.

Once a usable innovation is established, potential users can vary non-essential components to suit their circumstances. The realities of human services often require modifications in the delivery of services, and practitioners introduce their personality into service delivery. Fidelity data provide evidence to determine when modifications have “gone too far” and have compromised the essential components. As variations occur, maintaining the fidelity-outcome correlation is paramount so that benefits accrue to the intended population.

Fidelity is thoroughly embedded in the definition of any intervention that is evidence-based or scalable. Bond and Drake (100), pioneers in implementation research, note that, “Fidelity specification and measurement confer multifarious benefits to funders, program managers, clinicians, researchers, and patients. Without fidelity measures, treatment becomes a mysterious black box: We do not know precisely what the intervention is, how to implement it, and what quality of it has been delivered. The black-box approach represents pre-scientific clinical care. On the other hand, fidelity measurement provides clarity regarding the intervention model, its differentiation from other models, and its degree of implementation”.

At present fidelity assessments are missing or inadequate and, therefore, there is a notable lack of evidence that an independent variable is there. Consequently, the “science” in implementation science is not progressing as it might, and potential users are left wondering what to put in place to reliably produce promised benefits to people.

## Data Availability

The original contributions presented in the study are included in the article/Supplementary Material, further inquiries can be directed to the corresponding author.
